# Hydatid Disease With Psoas and Gluteal Muscle Involvement Causing Cauda Equina Compression: A Case Report

**DOI:** 10.7759/cureus.74029

**Published:** 2024-11-19

**Authors:** Anass Barchid, Achraf Laiz, Issam Yazough, Younes Aggouri, Said Aitlaalim

**Affiliations:** 1 General Surgery, Tangier University Hospital, Abdelmalek Essaâdi University, Tangier, MAR

**Keywords:** hydatid disease, psoas hydatid cyst, recurrent hydatid disease, soft tissue hydatid cyst, surgery

## Abstract

Hydatid disease is a zoonotic infection caused by the *Echinococcus granulosus *tapeworm, primarily affecting the liver and lungs, and rarely involving muscle tissue. Humans are infected by ingesting eggs from contaminated food or water. Patients may present with painless, slow-growing masses, sometimes associated with nerve or vessel compression, especially in cases of muscle involvement. Diagnostic imaging techniques such as ultrasonography, CT scan, and MRI are essential for preoperative evaluation. Treatment involves surgical removal of the cyst with careful lavage to prevent recurrence, followed by anti-parasitic medication. Our case involves a young female with a previously treated pulmonary hydatid cyst, who presented later with numbness and pain in her left lower limb, revealing a psoas location of hydatid disease exerting direct compression of the cauda equina. This case underscores the importance of including hydatid disease as a potential differential diagnosis for soft tissue masses in endemic areas, even with an atypical clinical presentation.

## Introduction

Hydatic cyst is an infection caused by a parasitic tapeworm, *Echinococcus granulosus*. It is primarily prevalent in pastoral areas [1]. Humans, as intermediate hosts, are infected through the ingestion of water or food contaminated by eggs from the feces of dogs [2]. The liver and lungs are the most frequently affected organs. Muscle involvement is rare, representing less than 0.9% of cases [1]. A painless, gradually enlarging swelling is the classical presentation of hydatid cysts in the extremities. Imaging remains essential for preoperative diagnosis, including ultrasonography, CT scan, and MRI to exclude other potential differentials [3].

Here, we report the case of multiple hydatid cysts involving both psoas and gluteal muscles, presenting with neurological symptoms in the lower limb, highlighting the unusual presentation and the importance of considering this disease, especially in endemic regions.

## Case presentation

We present the case of a 28-year-old female with a prior history of hydatid disease involving the lung, surgically removed in 2009. She reported no direct contact with dogs or wildlife. For the past four years, she has experienced numbness and pain in her left lower limb with increasing intensity, accompanied by swelling in her left gluteal region.

Physical examination revealed a noticeable swelling in the left calf, with tenderness. Laboratory values were normal, with hemoglobin at 13 g/dL, white blood cells at 6000/mm³, platelets at 284,000/mm³, liver enzymes within normal limits, and CRP at 2 mg/dL. Kidney function was also normal.

**Figure 1:**
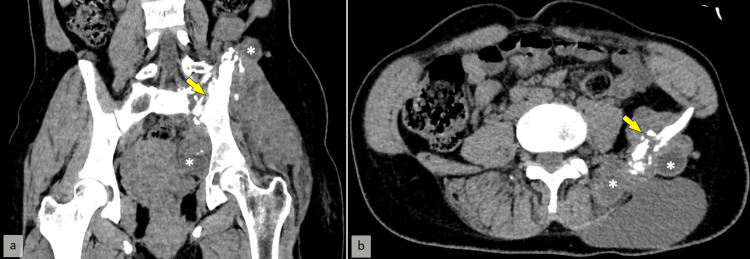
Coronal (a) and axial (b) CT scans show an enlarged left psoas muscle with a hypodense cystic lesion (*) and clear destruction of the left iliac wing (yellow arrow).

CT imaging showed a large multiloculated cystic mass in the left gluteal region, measuring 112 x 148 x 168 mm, causing bone lysis of the left iliac wing, the left transverse process of L5, and the left sacral wing, with extension into the spinal canal (Figure 1).

**Figure 2:**
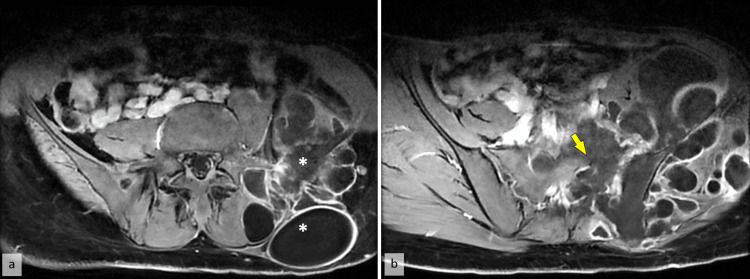
(a) Axial T2-weighted MRI scan of the pelvic region shows a hypointense lesion in the left psoas muscle, consistent with a fluid-filled hydatid cyst (*). (b) The cystic lesion causes destruction of the fifth lumbar vertebra and compression of the cauda equina (yellow arrow).

No thoracic lesions were observed. Pelvic MRI revealed a large, multiloculated cystic mass in the left gluteal region, measuring 112 x 148 x 168 mm. The radiological appearance was consistent with multiple hydatid disease locations. The cysts involved the left iliopsoas, gluteus minimus and medius, left piriformis, and paravertebral muscles, sparing the gluteus maximus, which showed atrophy. The lesion caused bone lysis of the left iliac wing, left transverse process of L5, and left sacral ala, with vertebral canal invasion and bilateral sacral foramina involvement. The cyst extended into the left L5 foraminal space, compressing the cauda equina roots at this level (Figure 2).

**Figure 3:**
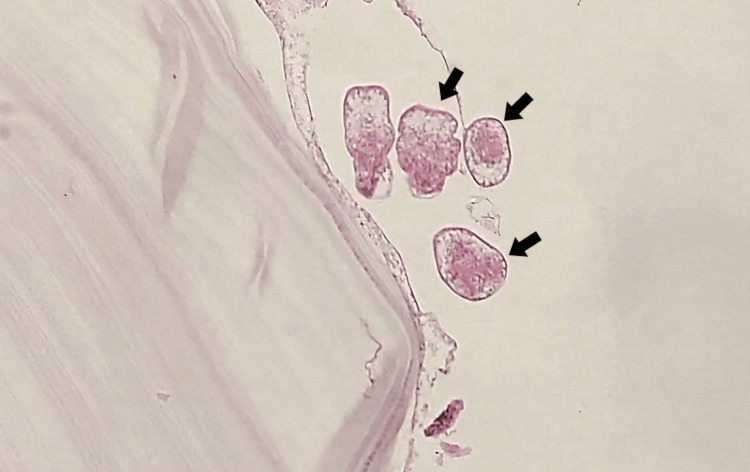
Pathologic examination of the cystic lesion shows multiple scolices (black arrows) and an acellular laminated membrane on the left (H&E, 400X). H&E: Hematoxylin and eosin stain

Complete cyst removal was achieved with an intact external capsule, followed by oral albendazole therapy (400 mg twice daily for six months). Pathological examination confirmed a hydatid cyst diagnosis (Figure 3). After one year, the patient showed partial recovery of symptoms, with a slight limp in her left leg.

## Discussion

Our patient presented with progressive left leg numbness and pain over four years, along with swelling in the left gluteal region. While this presentation strongly suggested a neoplastic origin, the patient's history of pulmonary hydatid disease raised the possibility of an unusual hydatid cyst location.

Hydatid disease is a zoonotic disease caused by *E. granulosus*, endemic in regions like the Mediterranean, the Middle East, and Morocco [4], particularly in pastoral areas. Ingestion of *Echinococcus* eggs results in oncospheres that travel via the bloodstream to various organs, most commonly the liver and lungs (accounting for nearly 90% of cases) [5], due to portal circulation. Rarely, these oncospheres bypass hepatic and pulmonary filters to reach other organs through systemic circulation [6]. Canines are definitive hosts, becoming infected by ingesting offal containing larval protoscoleces, which mature and release eggs in feces, subsequently infecting humans.

Hydatid cysts can occur in various organs, including the liver, lungs, kidneys, bone, and muscle tissue, with muscle involvement being rare [7]. It is important to consider hydatid disease in cases of soft tissue masses, particularly in endemic areas [8].

Soft tissue hydatid disease typically manifests as a gradually enlarging mass. Symptoms may include vessel or nerve compression [7] and, in some cases, superinfection or sepsis [9]. Our patient experienced neurological symptoms due to cauda equina compression, later accompanied by gluteal swelling. Serology can aid in diagnosis, but imaging is crucial for preoperative assessment. Ultrasound has a high diagnostic accuracy. CT allows precise anatomical assessment, and MRI, the most reliable tool, typically shows a multivesicular cyst with daughter cysts [10]. Histopathology is essential for postoperative confirmation. Differential diagnoses include a wide range of neoplastic and non-neoplastic conditions, such as soft tissue tumors, abscesses, and hematomas. These diagnoses can often be ruled out through imaging and further pathological analysis [7].

Surgery, involving careful removal of the cyst's outer capsule, is the treatment of choice for soft tissue hydatidosis, with lavage using hypertonic saline to prevent recurrence [7]. Postoperative albendazole therapy reduces recurrence risk [11]. In cases where surgery is unfeasible, scolicidal agents offer alternative management [10].

In our case, a gluteal muscle incision and left inguinal approach facilitated cyst removal, with thorough lavage using hydrogen peroxide. Another cyst in the left iliac muscle was also excised, with lavage and drainage.

Hydatid disease recurrence rates are approximately 10% [8]; complications may include anaphylaxis, as well as neurological and respiratory issues, depending on the location. Preoperative diagnosis is critical to prevent cyst rupture and dissemination. In this case, a previous lung hydatid cyst raised the possibility of preoperative dissemination; however, primary disease could not be excluded.

## Conclusions

This case highlights the atypical manifestation of hydatid disease in muscle tissue, presenting with neurological symptoms from cauda equina compression. Despite its rarity, hydatid cysts should be included in the differential diagnoses of soft tissue masses. Imaging techniques, including CT and MRI, are critical for preoperative diagnosis and surgical planning. Complete cyst excision and anti-parasitic therapy are essential for minimizing recurrence, along with postoperative follow-up for early detection and effective treatment strategies.
